# Advanced Design Strategies for Stable Sodium Metal Anodes: A Review

**DOI:** 10.3390/molecules31183158

**Published:** 2026-09-08

**Authors:** Jiaoli Gu, Hao Zhu, Zihao Bian, Dan Nie, Jiaojiao Li, Anlin Zhang, Xianming Xia, Hang Zhang, Bin Deng, Ruijin Yu

**Affiliations:** 1 College of Chemistry and Environmental Science, Xiangnan University, Chenzhou 423000, China; 2 Jiangxi Province Key Laboratory of Porous Functional Materials, College of Chemistry and Materials, Jiangxi Normal University, Nanchang 330022, China; 3 Hunan Provincial Key Laboratory of Xiangnan Rare-Precious Metals Compounds Research and Application, Xiangnan University, Chenzhou 423000, China; 4 College of Chemistry & Pharmacy, Northwest A&F University, Yangling 712100, China

**Keywords:** sodium metal anodes, dendrite suppression, current collector engineering, electrolyte optimization, artificial SEI, interfacial stability

## Abstract

Sodium metal anodes (SMAs) are regarded as the most promising anode materials for next-generation high-energy-density sodium metal batteries, owing to their ultrahigh theoretical specific capacity (1166 mAh g^−1^) and low electrochemical potential (−2.71 V vs. SHEs). However, their practical application is severely hindered by a series of interrelated challenges, including unstable solid electrolyte interphase (SEI) films, severe volume fluctuations arising from their hostless nature, uncontrollable dendrite growth, and the consequent low Coulombic efficiency and short cycle life. This review systematically summarizes recent progress in stabilizing SMAs through three major categories of strategies: current collector engineering, which involves the design of planar, three-dimensional, and gradient architectures to regulate the local current density and Na^+^ flux, thereby guiding uniform nucleation and enabling “bottom-up” dendrite-free deposition; electrolyte engineering, which focuses on optimizing solvents, salts, and functional additives to tailor the solvation structure, construct robust inorganic-rich SEI layers, and utilize electrostatic shielding effects to suppress dendrite formation; and artificial SEI engineering, which aims to pre-construct inorganic or inorganic–organic hybrid protective layers that establish a physicochemical barrier between the electrode and electrolyte, combining high ionic conductivity, superior mechanical strength, and sufficient flexibility. Finally, we provide a critical perspective on the remaining challenges and outline future research directions, emphasizing the importance of in situ/operando characterization, synergistic multi-strategy integration, breakthroughs in high areal capacity and high-rate performance, and artificial intelligence-driven material discovery for the practical implementation of SMAs.

## 1. Introduction

The escalating global energy crisis and the pressing demand for environmental sustainability have driven advancements in high-energy-density and cost-effective electrochemical energy storage technologies. Lithium-ion batteries, which currently dominate the portable electronics and electric vehicle markets, face significant challenges, including rising lithium resource costs, uneven geographical distribution, and increasing safety concerns [1,2,3]. Consequently, exploring alternative energy storage systems that can meet the growing demand for energy density while ensuring economic viability has become a critical research priority. In this context, sodium-ion batteries have emerged as a highly promising next-generation energy storage technology, primarily due to the natural abundance of sodium (Na) resources and their cost-effectiveness [4,5,6,7]. More importantly, Na metal anodes (SMAs), which possess a high theoretical specific capacity of 1166 mAh g^−1^ and low electrochemical potential (−2.71 V vs. standard hydrogen electrodes (SHEs)), are regarded as the ultimate anode materials for achieving high-energy-density Na metal batteries (SMBs) [8,9,10,11]. For perspective, the theoretical capacity of Na is approximately three times that of conventional graphite anodes (372 mAh g^−1^) used in lithium-ion batteries and is substantially higher than that of typical intercalation-type anodes, while its electrochemical potential is close to that of lithium (−3.04 V vs. SHEs). This combination of high capacity and low potential underpins the exceptional energy density potential of SMAs, making them a compelling candidate for next-generation energy storage systems. Leveraging these exceptional properties, the energy density of SMBs can potentially approach or even exceed that of conventional lithium-ion batteries, offering a viable pathway for large-scale energy storage applications.

Despite these inherent advantages, the practical implementation of SMAs is significantly hindered by a series of fundamental challenges arising from the intrinsic chemical and physical properties of Na metal [12,13]. These challenges are primarily manifested in four interconnected aspects [13,14,15,16]. First, the exceptionally high reactivity of Na metal with organic electrolytes leads to the continuous formation and evolution of an unstable solid electrolyte interphase (SEI) film. The high solubility of SEI components, combined with the dynamic rupture–regeneration processes occurring during cycling, results in the persistent consumption of active Na and electrolyte, ultimately causing capacity degradation and diminished coulombic efficiency (CE). Second, SMAs are inherently hostless systems that lack a physical support framework during deposition. This characteristic results in substantial and nearly infinite volume fluctuations during plating/stripping cycles, inducing structural collapse, electrode pulverization, and the generation of electrochemically inert “dead Na”. Such morphological deterioration severely compromises the structural integrity and reversibility of the anode. Third, the non-uniform nucleation and growth of Na dendrites, driven by the uneven distribution of ion flux and electric fields, represents one of the most hazardous failure modes. The soft mechanical properties of Na metal further exacerbate the risk of dendrite penetration through the separator, leading to internal short circuits and potential thermal runaway. Fourth, these intertwined issues collectively contribute to low CE and a limited cycle life, creating a fundamental dilemma wherein the pursuit of high energy density is inherently incompatible with long-term cycling stability.

**Figure 1:**
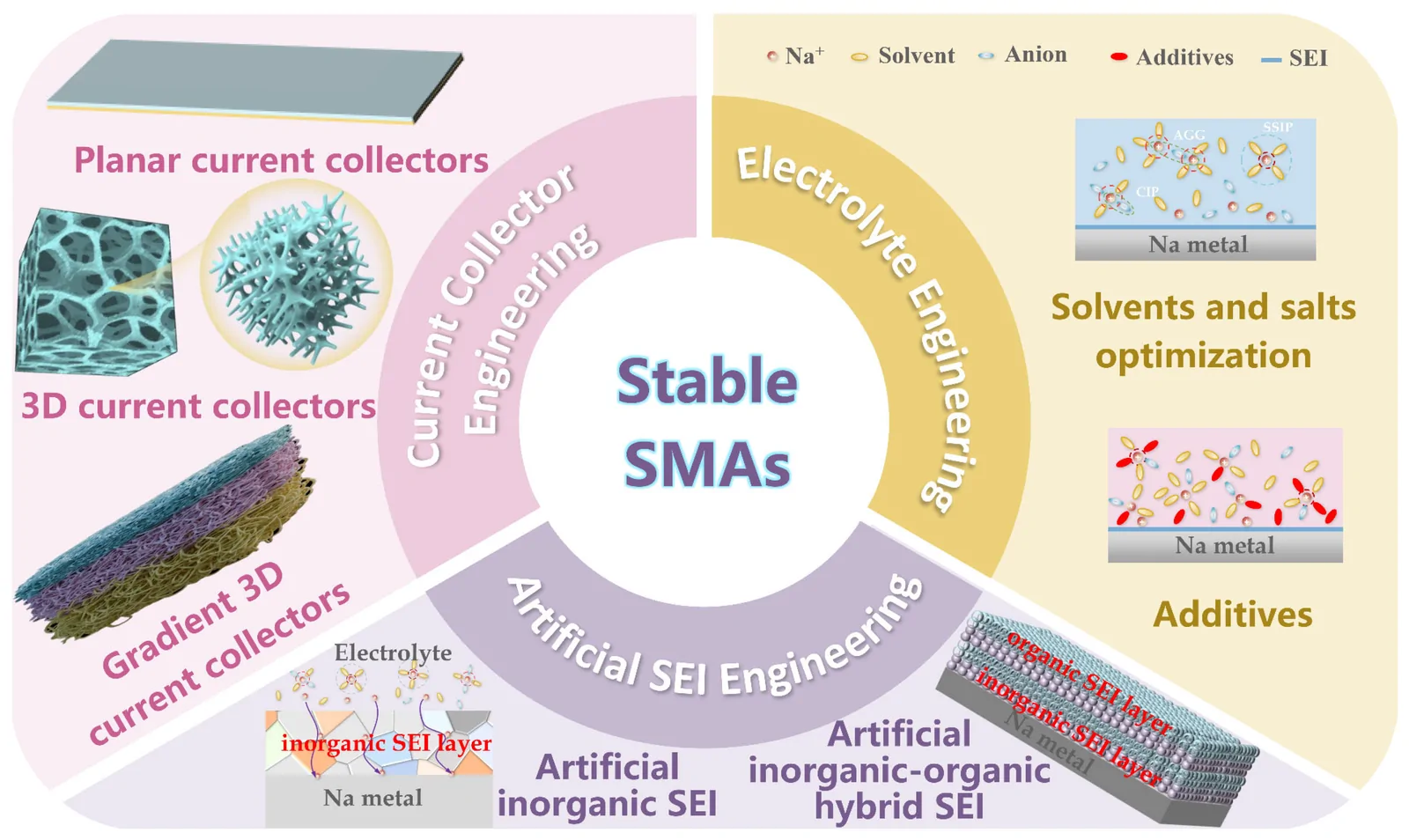
Schematic illustration of the differently improved engineering strategies for stable SMAs.

To address these multifaceted challenges, extensive research efforts have been devoted over the past decade to developing innovative strategies for stabilizing SMAs [17,18,19]. These strategies aim to regulate the sodium deposition behavior, enhance the interfacial stability, and mitigate volume expansion, thereby improving the electrochemical performance and safety of SMBs [8,20,21,22,23,24,25]. This review systematically summarizes the recent progress in enhancing the performance of SMAs (Figure 1), focusing on three major categories of strategies: current collector engineering, which involves designing planar, three-dimensional (3D), and gradient architectures to guide uniform Na nucleation and deposition; electrolyte engineering, which encompasses the optimization of solvents, salts, and functional additives to tailor the solvation structure and construct a robust and stable SEI; and artificial SEI engineering, which includes the fabrication of inorganic and inorganic–organic hybrid protective layers that synergistically combine high ionic conductivity, mechanical strength, and flexibility. By providing a comprehensive overview of these state-of-the-art approaches, this review aims to elucidate the underlying mechanisms governing the performance of SMAs and offer critical insights for the rational design of high-performance, dendrite-free, and long-life SMBs. Finally, we provide a critical perspective on the remaining challenges and outline future research directions for practical applications.

## 2. Challenges of Sodium Metal Anodes

Although SMAs are regarded as ideal materials for constructing next-generation sodium-based batteries with high energy density, they encounter multiple challenges in practical applications [26,27,28,29]. These challenges arise from the intrinsic properties of the metallic Na and the coupling of electrochemical processes. They are primarily manifested as unstable SEI films, significant volume changes, uncontrollable dendrite growth, and the resulting issues of low CE and a poor cycle life. A comprehensive understanding of these challenges is essential in developing effective strategies for stable SMAs.

### 2.1. Instability of Interfacial Chemistry: Formation and Evolution of SEI

When metallic Na contacts the electrolyte, its exceptionally high reactivity triggers immediate side reactions, resulting in the formation of a passivation layer known as the SEI film. This film should ideally exhibit uniformity, density, high ionic conductivity, mechanical flexibility, and electrochemical stability to prevent ongoing side reactions and facilitate uniform sodium deposition [30,31]. However, the SEI films on SMAs present two critical challenges. First, they possess intrinsic instability and high solubility. In comparison to lithium, sodium exhibits weaker Lewis acidity, which leads to SEI components, such as sodium salts and alkyl carbonates, that are formed from electrolyte decomposition products with higher solubility in conventional electrolytes. Consequently, the SEI film continuously dissolves during cycling, failing to provide long-term passivation and exposing fresh sodium metal surfaces, thereby triggering further side reactions. Second, there is the issue of dynamic rupture and regeneration. Na metal undergoes nearly infinite volume changes during deposition and stripping processes, and the SEI film is unable to withstand such severe deformation, resulting in rupture as the metal matrix expands [13,32]. This dynamic process of “rupture–regeneration” continuously consumes active sodium and electrolyte, leading to the increased thickness of the SEI film and a persistent rise in impedance, ultimately causing rapid capacity decay in the battery.

### 2.2. Hostless Nature: Volume Effects and Structural Failure

Unlike embedded anodes with stable lattice structures, such as hard carbon, SMAs lack a physical support framework during the deposition process, rendering them a typical “hostless” system. During deposition, sodium atoms nucleate and grow on the surface of the current collector, with their volume expanding from zero to the total volume of deposited sodium. Conversely, during stripping, the deposited sodium nearly completely dissolves, leaving behind voids or isolated structures [14]. This “hostless” characteristic gives rise to two significant issues. Firstly, the macroscopic structure of the electrode collapses. Over prolonged cycling, repeated volume changes can cause the sodium metal electrode to transition from a dense structure to a porous, powdered form [13]. This structural evolution not only increases the specific surface area of the electrode, exacerbating the formation of the SEI film, but may also lead to the partial delamination of the sodium metal from the current collector, resulting in electrochemically inert “dead sodium”, which directly contributes to a loss in reversible capacity. Secondly, interfacial contact deteriorates. The drastic volume fluctuations hinder the stability of the solid–solid interfaces between the sodium metal, the separator, and the SEI, and poor contact can lead to the further concentration of the local current density, creating new hotspots for dendrite growth.

### 2.3. Uncontrollable Dendrite Growth: Thermodynamic and Kinetic Mechanisms

Dendrite growth represents one of the most common and hazardous manifestations of SMA failure, fundamentally arising from non-equilibrium nucleation and growth induced by uneven ion flux and electric field distribution during the deposition process. From a thermodynamic perspective, the nucleation energy barrier for sodium ions on the electrode surface during the early stages of electrochemical deposition is critical in determining the uniformity of deposition. Due to the inevitable presence of defects, impurities, or protrusions in the SEI film on the sodium metal surface, these sites exhibit lower nucleation overpotential, thereby becoming preferential growth locations [33]. From a kinetic standpoint, once initial protrusions form, the local current density at the tips of these protrusions becomes significantly higher than that in the flat regions. According to Sand’s time model, when the local current density surpasses the limiting diffusion current density for ion transport within the electrolyte system, an ion depletion layer emerges, exacerbating the concentration gradient and distorting the electric field at the tip. This creates a positive feedback mechanism that propels dendrite growth in the direction of the electric field [34]. Furthermore, the soft mechanical properties of sodium metal further amplify the hazards associated with dendrite formation. Sodium possesses a low Young’s modulus (approximately 10 GPa) and a soft texture, which facilitate the penetration of dendrites through the separator with minimal mechanical resistance [35,36]. Concurrently, under the pressure of battery assembly, the sodium anode is susceptible to creep, leading to its intrusion into the pores of the separator and heightening the risk of internal short circuits. Dendrite growth not only results in the puncturing of the separator, which can trigger thermal runaway, but also induces fractures at the base of the dendrites due to the concentrated current, leading to the formation of “dead sodium” that loses electrical contact with the current collector, thereby directly causing a loss of reversible capacity [37].

### 2.4. Low CE and Short Cycle Life

CE is a crucial electrochemical metric for assessing the reversibility of sodium metal anodes. Ideally, sodium metal anodes should achieve CE close to 100%, indicating that nearly all deposited sodium can be reversibly utilized during subsequent stripping processes. However, due to the continuous formation and evolution of the SEI film, dendrite growth, and the accumulation of “dead sodium”, sodium metal anodes typically exhibit low CE during actual cycling. The primary reason for low CE is the irreversible loss of active sodium, which arises from two main factors. The first is the ongoing formation and repair of the SEI film. Upon contact with the electrolyte, sodium metal immediately undergoes side reactions that generate an SEI film. During cycling, volume changes can cause the SEI film to crack, continuously exposing fresh sodium surfaces and initiating new side reactions, which persistently consume active sodium and electrolyte [31,38]. The second is the accumulation of “dead sodium”. Dendrites formed during deposition are prone to fracture during stripping, losing electrical contact with the current collector and resulting in electrochemically inert “dead sodium” [37]. As the number of cycles increases, “dead sodium” gradually accumulates, leading not only to a continuous decline in CE but also to an increase in the porosity and interfacial impedance of the electrode. There exists a direct causal relationship between low CE and a short cycle life. In button cell half-battery tests, even with single-cycle CE of 99%, the cumulative loss of active sodium after 100 cycles can reach approximately 63%. In the design of full batteries intended for practical applications, the pursuit of high energy density often necessitates strict control over the amount of sodium in the anode (low N/P ratio). This low CE results in the rapid depletion of limited Na reserves, leading to a significant decline in battery capacity within just a few dozen cycles. This situation presents a fundamental contradiction faced by SMAs: on one hand, the high theoretical specific capacity of sodium metal (1166 mAh g^−1^) provides the physical basis for achieving high-energy-density batteries; on the other hand, the existence of low CE necessitates the excessive use of Na metal (high N/P ratio) to achieve an acceptable cycle life. Consequently, the actual capacity contribution of the anode falls far below its theoretical value, severely diluting the overall energy density and creating a dilemma wherein high energy density and a long cycle life cannot coexist [39].

## 3. Strategies for Stable SMAs

With the continuous advancement of characterization techniques and theoretical simulations, researchers have gained a deeper understanding of SMAs. To facilitate the practical application of highly stable and safe SMBs, numerous researchers have proposed and designed various strategies to optimize and enhance SMAs, with the goal of improving their electrochemical performance. In this paper, we summarize several strategies for enhancing the performance of SMAs, including current collector engineering, electrolyte engineering, and artificial SEI engineering.

### 3.1. Current Collector Engineering

Current collectors are a crucial component of SMAs, serving not only as a substrate for Na metal deposition but also influencing the deposition behavior of Na metal and dendrite formation, which are vital for battery safety and its cycle life [40,41,42]. Therefore, the rational design of the surface structures and functional components of current collectors to regulate Na deposition behavior is one of the most important strategies for achieving dendrite-free and stable SMAs. This section categorizes current collectors into three major types based on their structural and functional differences: planar current collectors, 3D current collectors, and gradient 3D current collectors.

#### 3.1.1. Planar Current Collectors

The morphology of Na deposition is intricately related to the initial nucleation behavior of Na^+^. When Na^+^ is deposited on the surfaces of traditional two-dimensional current collectors, the unevenness of the surface leads to non-uniform charge distribution, which results in the formation of Na dendrites induced by inhomogeneous Na deposition [43]. Recently, numerous researchers have introduced a “sodiophilic” layer to regulate the deposition behavior of Na^+^ on current collectors, thereby achieving uniform, dendrite-free, and stable SMAs [44,45]. For instance, Tang et al. designed a current collector for SMAs via magnetron sputtering, depositing a thin and uniform gold layer on the surface of Cu foil (Cu@Au) [46]. Their experimental findings revealed the presence of the Au_2_Na phase after the initial electroplating, indicating that an alloying reaction occurred between Au and Na. Furthermore, they discovered that, during the electroplating process, Na^+^ exhibited a low nucleation overpotential on Cu@Au, which is advantageous for achieving uniform Na deposition. Theoretical calculations also suggested that the binding energy between Cu and Na is relatively low, resulting in a high nucleation barrier for Na deposition on Cu, leading to uneven Na deposition. In contrast, the Au-Na alloy layer possessed a higher binding energy with Na, significantly increasing the number of nucleation sites for Na deposition, ultimately resulting in a uniform and dense Na layer. Moreover, the optical photographs and scanning electron microscopy (SEM) images obtained after plating/stripping cycling further demonstrated that Cu@Au could effectively regulate Na deposition, achieving uniform, dense, and dendrite-free SMAs. However, during the plating/stripping process, the materials continuously undergo alloying and dealloying, which undoubtedly damages the sodiophilic layer, leading to the loss of its original capability to regulate Na^+^ nucleation behavior, thereby limiting the cycling life of the SMA [47,48,49]. Innovatively, a recent study conducted by Mao’s group utilized sodiophilic materials (such as Sn and Sb) in conjunction with precise control over the cutoff potential [50]. This approach effectively prevented the dealloying process, thereby preserving the integrity of the highly sodiophilic M-Na alloy (M = Sn, Sb, etc.) layer. Consequently, this innovation significantly enhanced the stability of the plating/stripping processes, achieving up to 2000 (Sn) and 1700 (Sb) cycles at 2 mA cm^−2^.

To address the issue of volume changes during the alloying–dealloying process that lead to the loss of the original functionality in materials, a common strategy employed in ion batteries is the carbon coating of active materials [51,52]. This concept has also been applied to protect the relevant properties of sodiophilic materials. For instance, Wang et al. proposed a simple and scalable method for synthesizing Sn nanoparticles (NPs) uniformly embedded in a carbon network (Sn@C), which are coated on Cu foil to serve as current collectors for SMAs [53]. Their study revealed that Sn NPs can act as preferential nucleation sites for Na^+^, guiding its nucleation and thereby reducing the overpotential for Na deposition. The carbon network functions as a buffering layer, effectively mitigating volume changes and reducing the shedding of Sn NPs during repeated plating/stripping cycles. Furthermore, ex situ SEM images indicate that the surface of the Sn@C current collector exhibits a smooth and dendrite-free morphology after cycling, demonstrating that Sn@C can effectively regulate the plating/stripping process of Na. Similarly, Wu’s group designed a current collector modified by an innovative nucleation layer composed of C@Sb NPs that incorporated a meticulously designed core–shell configuration aimed at spatially controlling Na metal deposition [54]. This distinctive structural approach enables both internal and external elements to utilize their individual strengths. The Sb cores, which are porous, showcase a substantial surface area along with a notably low nucleation barrier for Na^+^, thereby offering numerous initial nucleation sites. The carbon shells on the exterior serve to avert the agglomeration of the porous Sb NPs throughout the cycling process, thus maintaining the efficiency of the Sb NPs for extended durability. As a result, cells employing C@Sb-coated Cu foil as electrodes exhibited cycling stability of nearly 6000 and 2600 h at 4 and 6 mAh cm^−2^, respectively. Furthermore, differing from the carbon coating strategy, Lu et al. reported hierarchical Sb_2_MoO_6_ (SMO) microspheres assembled by one-dimensional nanobelts, which, when coated onto Cu foil, served as a current collector, enabling high-performance SMAs [55]. In the design, the SMO substrate can self-assemble into a structure comprising a “seed (Sb)-embedded conductive buffer matrix (Na-Mo-O)” prior to the Na deposition process. The Sb NPs serve as sodiophilic “nanoseeds” that facilitate Na nucleation due to the tendency of Sb to alloy with Na, thereby reducing the Na nucleation potential and promoting uniform Na deposition. Moreover, the conductive Na-Mo-O buffer matrix effectively mitigates the volume changes of the Na-Sb alloys during repeated Na plating/stripping processes. Benefiting from these merits, the symmetrical cells assembled with Na-pre-deposited SMO electrodes exhibited stable cycling for over 500 cycles at 10 mA cm^−2^ with 8 mAh cm^−2^. Impressively, Fang’s group recently reported a CeF_3_@NC-modified current collector for stable SMAs [56]. They confirmed that the conversion reaction between Na^+^ and CeF_3_@NC formed NaF and metallic Ce sites during the initial cycling. The well-defined NaF-rich SEI ensures interfacial stability even under a high depth of discharge. Moreover, the in situ-formed metallic Ce sites exhibit notable attraction to PF_6_^−^ anions, which enhances the desolvation of Na^+^ by reducing the energy barrier involved in the progressive extraction of anions. Additionally, this interaction plays a crucial role in modulating the distribution of ions at the interface, encourages the accumulation of PF_6_^−^ anions at the inner Helmholtz plane, and supports the development of a dynamically self-repairing interphase that ensures long-term stability. As a result, the obtained SMAs achieved stable operation for over 5800 h.

**Figure 2:**
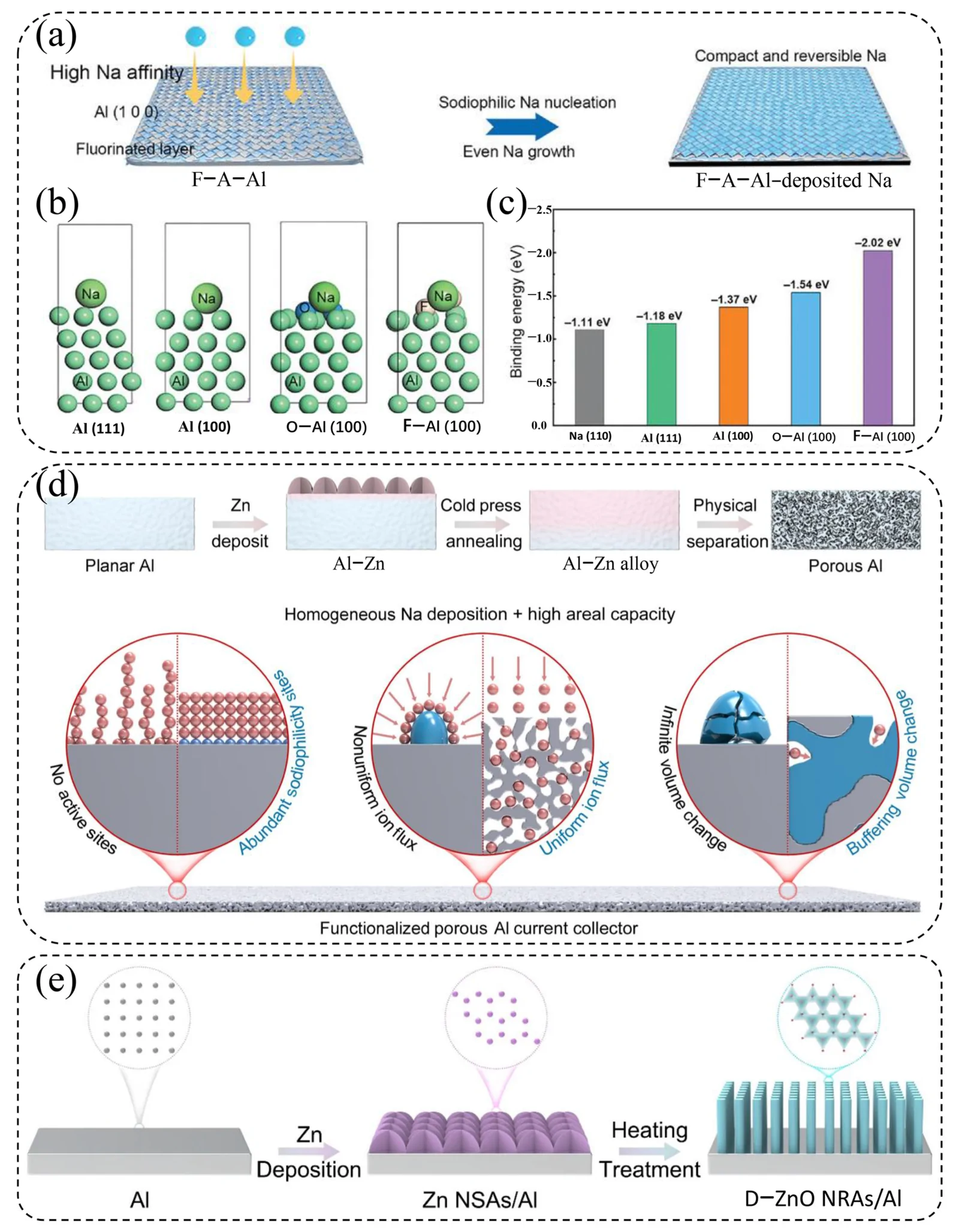
(**a**) Schematic illustration of F−A−Al regulating Na deposition. (**b**,**c**) The adsorption model of Na atoms on different substrates and the relative binding energies [58]. (**d**) Schematic showing the synthesis process and main features of the 3D porous Al collector [65]. (**e**) Schematic drawing of the fabrication process of D-ZnO NRAs/Al [73].

Beyond the aforementioned strategies, regulating the structure and functional composition of planar current collectors is also an effective strategy to regulate the Na deposition behavior and achieve stable SMAs. For instance, Qiao’s group designed a grain-optimized Cu current collector [57]. They systematically investigated how the grain boundary density influenced the interfacial electrochemical behavior of Na^+^ and anions. Their findings indicate that Cu with a greater density of grain boundaries shows stronger attraction to Na, which can reduce the energy barrier for Na nucleation, thereby promoting the development of highly crystalline and closely packed Na deposits. Additionally, the elevated surface energy at the grain boundaries enhances anion adsorption, resulting in a solvation structure at the interface that is rich in anions, ultimately leading to the formation of a stable and thin NaF-rich SEI. Therefore, the Cu current collector with an ultrahigh grain boundary density exhibited high Na plating/stripping CE of 99.98% at 1 mA cm^−2^. Moreover, as shown in Figure 2a, Wu et al. reported a novel Al current collector with a controllable crystal orientation and surface properties via annealing and fluorination processes (the obtained current collector is named “F-A-Al”) [58]. Density functional theory (DFT) revealed that the fluorinated Al (100) planes exhibited strong binding energies for Na atoms (−2.02 eV), in contrast to those of Al (111) (−1.18 eV), Al (100) (−1.37 eV), and oxidized Al (100) (−1.54 eV) (Figure 2b,c). This confirmed that the fluorinated Al (100) planes had high sodiophilicity, which enabled homogeneous Na nucleation and deposition. Moreover, the optimized Al current collector promoted the formation of robust SEI layers rich in inorganic NaF and FSA^−^-based species, which enhanced the CE and cycle life of the obtained SMB.

Although the aforementioned research has contributed to the achievement of uniform Na^+^ nucleation, enabling the development of dendrite-free SMAs, the capacity for Na deposition remains relatively limited, and this limitation poses challenges in attaining high-capacity SMBs. Consequently, there is an urgent need to develop and investigate more distinctive current collectors for SMAs.

#### 3.1.2. 3D Current Collectors

Compared to planar two-dimensional current collectors, 3D structures can effectively increase the specific surface areas of the electrodes. This characteristic reduces the local current density and promotes the more uniform distribution of Na^+^. Moreover, it provides more active sites for initial Na^+^ nucleation and can delay the formation time of Na dendrites to some extent [14,59]. Therefore, the construction of 3D current collectors is more conducive to enhancing the electrochemical performance of SMAs.

Metal-based current collectors possess advantages such as high electrical conductivity, high mechanical strength, and ease of manufacturing, making them the subject of extensive research among scholars [40,41,60,61]. Recently, Sun et al. reported that 3D porous Cu significantly enhanced the stability of Na plating/stripping processes, achieving high CE of 99.4% after 400 cycles at 1 mA cm^−2^. This improvement is attributed to the large specific surface area of the 3D porous Cu current collector, coupled with its excellent wettability with the electrolyte, which contributed to lowering the local current density and promoting dendrite-free growth [62]. Moreover, Wang et al. designed a porous Cu foam current collector modified by Cu nanowires (CuNW-Cu) via an in situ surface treatment method [63]. The Cu nanowires significantly increased the specific surface area of CuNW-Cu, which not only provided uniform Na^+^/electron diffusion pathways and ample nucleation sites but also reduced the local current density. More importantly, as a high-strength 3D support, CuNW-Cu was able to confine the deposited Na within its nanowire-enhanced regions, effectively suppressing the growth of Na dendrites. Owning to its unique structure, the CuNW-Cu current collector exhibited high reversible Na plating/stripping performance of over 1400 h at 1.0 mA cm^−2^ with 2.0 mAh cm^−2^. Compared with Cu current collectors, aluminum (Al) offers several benefits, including being lightweight, readily available, and cost-effective, indicating a promising future for its use in SMA current collectors. (Rapid and reversible Na deposition onto Al nanosheet arrays) For instance, Luo et al. reported a 3D porous Al current collector for stable SMAs [64]. In this design, the porous interconnected framework can enhance the accessible surface area for Na nucleation and regulate the distribution of Na^+^ flux, resulting in uniform Na plating. Consequently, the 3D porous Al current collector displayed stable Na plating/stripping cycling for over 1000 cycles, with minimal and stable voltage hysteresis and high average CE of 99.9%, marking a significant improvement over planar Al current collectors. Similarly, Lou’s group meticulously designed a functionalized 3D porous Al current collector via template-free electrodeposition and physical separation techniques (Figure 2d) [65]. The architecture, with controlled porosity, which includes networks of interconnected ligament channels, diminishes structural stress and prevents the formation of dendritic Na by decreasing the local current density and promoting the uniform distribution of ion flux. Furthermore, the plentiful sodiophilic active sites enhance the kinetics of the reaction, lower the nucleation barrier for Na^+^, and subsequently facilitate the uniform nucleation of Na. Therefore, the 3D porous Al current collector delivered average CE of 100% at 10 mA cm^−2^ with 20 mAh cm^−2^ over 900 h.

Beyond the aforementioned reports on 3D metal-based current collectors, many efforts have focused on modifying metal-based current collectors by introducing highly sodiophilic components, with the aim of regulating the Na deposition behavior to achieve high-performance SMAs without dendrites [66,67]. Recently, Wang et al. created a novel 3D porous Cu foam current collector decorated with a gradient heterostructure composed of CuSn alloy and metallic Sn through a simple electroless plating method [68]. In the design, the CuSn alloy interlayer mitigates volume expansion and diffuses mechanical stress during cycling, while the outer Sn layer offers numerous low-barrier nucleation sites that ensure uniform and dense Na deposition within the 3D porous structure. Consequently, the 3D current collector delivered high performance and stable CE; meanwhile, the symmetrical cell coupled with the pre-deposited Na 3D current collector exhibited superior plating/stripping cycling over 2000 h at 1 mA cm^−2^ with 1 mAh cm^−2^. In addition to such simple modifications, the construction of sodiophilic array structures can not only provide more nucleation sites for Na^+^ but also regulate the distribution of the electric fields and ionic flux, promoting uniform nucleation and deposition [69,70,71]. For example, Yang et al. developed a Cu foam modified with hierarchical ZnO nanorod arrays that served as a 3D current collector (CF@ZnO) [72]. Compared to pure 3D Cu foam current collectors, this unique 3D CF@ZnO current collector features high sodiophilicity due to the presence of ZnO nanorods, which create a substantial number of Na^+^ nucleation sites, effectively reducing the Na nucleation overpotential and the local effective current density. Moreover, COMSOL Multiphysics (5.4) simulations confirm that the vertically grown layered ZnO nanorod array can also adjust the electric field distribution, thereby promoting the uniform deposition of Na^+^ on the CF@ZnO. As a result, the 3D CF@ZnO current collector exhibited small Na nucleation overpotential and stable Na plating/stripping operation (over 300 cycles) at 1 mA cm^−2^ with 1 mAh cm^−2^. Recently, Lou’s group systematically investigated the influence of sodiophilic arrays in improving electrochemical performance [73]. In their design, a lightweight Al foil serves as a substrate for the in situ growth of dense ZnO nanorod arrays (D-ZnO NRAs/Al) through electrodeposition, followed by low-temperature heat treatment processes (Figure 2e). The DFT results indicate that this elaborate current collector design possesses a high binding energy, indicating high sodiophilicity. The low nucleation overpotential further highlights the excellent sodiophilicity of the D-ZnO NRA/Al system. Furthermore, simulation results reveal that the D-ZnO NRA/Al current collector is expected to regulate the distribution of the electric field, current density, and electrolyte concentration, thereby facilitating outstanding electrochemical performance. Owing to these merits, the D-ZnO NRA/Al current collector displayed enhanced Na plating/stripping cycling stability (over 1500 cycles) with high average CE of 99.95% at 1 mA cm^−2^ and 1 mAh cm^−2^.

Compared with metal-based current collectors, carbon-based materials have been widely used in the field of energy storage due to their excellent chemical stability, low cost, and abundant availability [74,75]. Recent studies have confirmed the feasibility of constructing 3D carbon-based current collectors for regulating the uniform deposition of Na [76,77,78]. For instance, Zhang and his colleagues proposed carbon fiber paper (CFP) as a current collector for SMAs [79]. Its high sodiophilicity and unique 3D structure facilitate the uniform deposition of Na in 3D space, effectively preventing the formation of dendrites. Furthermore, during the repeated plating/stripping of Na in the CFP current collector, a low hysteresis voltage and excellent electrochemical stability were observed. Regrettably, the sodiophilicity of this pure carbon 3D current collector is limited due to the relatively high nucleation barrier for Na^+^. To enhance the sodiophilicity and achieve uniform Na deposition, Liu et al. adopted the strategy of doping a carbon framework with heteroatoms [80]. They constructed a nitrogen-doped porous carbon fiber framework as a 3D current collector for SMAs using electrospinning technology. DFT calculations indicate that the binding energy of Na^+^ with nitrogen-doped carbon (−0.83 eV) is significantly stronger than that of pure carbon (−0.48 eV), suggesting that nitrogen doping can enhance the adsorption of Na^+^, facilitating subsequent uniform Na deposition. Moreover, symmetric cells assembled with pre-deposition Na nitrogen-doped carbon fiber electrodes exhibited stable cycling performance for 3500 h and 950 h at 2 mA cm^−2^ and 4 mA cm^−2^, respectively. This exceptional electrochemical performance can be attributed to its rich porous structure, which mitigates volume changes during the plating/stripping processes, thus maintaining consistent performance throughout cycling. Moreover, Chu et al. introduced a nitrogen and oxygen co-doping strategy to improve the sodiophilicity of a carbon nanofiber network-wrapped carbon felt (NO-CNCF) [81]. DFT simulations revealed that the uniformly distributed N- and O-containing functional groups presented high binding energies between Na atoms and NO-CNCF, indicating high sodiophilicity, which reduced the energy barriers and facilitated the preferential deposition of Na^+^ on NO-CNCF. Furthermore, finite element simulations confirmed that NO-CNCF, characterized by evenly distributed sodiophilic sites and multistage structures, regulated the electric field and current density distribution, facilitating the consistent nucleation and deposition of Na. Benefiting from its structural features, the NO-CNCF system achieved stable cycling for over 4000 h at 1 mA cm^−2^ with 1 mAh cm^−2^ in symmetric cells.

Moreover, similarly to the strategies employed for modifying metal-based current collectors by introducing highly sodiophilic components, considerable efforts have been made to modify carbon-based current collectors to regulate the Na deposition behavior, thereby achieving stable and dendrite-free SMAs [82,83]. For example, Li’s group developed a self-supporting current collector consisting of ultrafine Sb_2_S_3_ nanoparticles encapsulated within carbon nanofibers (Sb_2_S_3_@CNFs), using electrospinning methods followed by sulfurization [84]. In the novel design, the nanoparticles of Sb_2_S_3_ demonstrate a greater affinity for Na, a phenomenon attributed to a conversion–alloying reaction with Na^+^, which additionally lowers the Na nucleation overpotential. Furthermore, the in situ-generated Na_2_S matrix features an exceptionally low diffusion barrier for Na, creating swift transport pathways for Na^+^/electronic alongside the carbon nanofibers that facilitate charge transfer. Consequently, the distinct spatially constrained “sodiophilic–conductive” network allows for uniform Na deposition and mitigates structural failure resulting from the expansion of the Na volume. Consequently, the Na deposition behavior could be regulated, enabling stable plating/stripping cycles exceeding 1000 cycles at 2 mA cm^−2^. Moreover, Guo et al. fabricated a self-supporting current collector composed of nitrogen-doped carbon hollow nanofibers with uniformly embedded MgF_2_ (MgF_2_@NCHNFs) [85]. In the initial Na plating process, the MgF_2_ undergoes in situ transformation into a gradient fluorinated alloy structure, where the outer layer of NaF assists in equalizing the Na^+^ flux, while the gradient arrangement of Mg and the subsurface sodiophilic N sites facilitate the deposition of Na within the internal spaces of the hollow nanofibers.

#### 3.1.3. Gradient 3D Current Collectors

Although traditional 3D current collectors provide a significant specific surface area and a porous structure that alleviates volume changes during Na plating/stripping, as well as reducing the local current density to some extent, they still encounter numerous challenges in practical applications. Concentrated electric fields and shorter ion transport pathways cause Na^+^ ions to be preferentially reduced and deposited on the side of the current collector facing the separator, resulting in a “top-down” growth mode [86,87,88]. This phenomenon leads to the poor utilization of the internal space of the 3D current collector, with dendrites easily forming on the surface and penetrating the separator [8,41]. In this context, constructing gradient structures along the thickness direction of the current collector has emerged as an effective strategy for inducing uniform “bottom-up” Na deposition, thereby achieving dendrite-free, high-areal-capacity SMAs.

**Figure 3:**
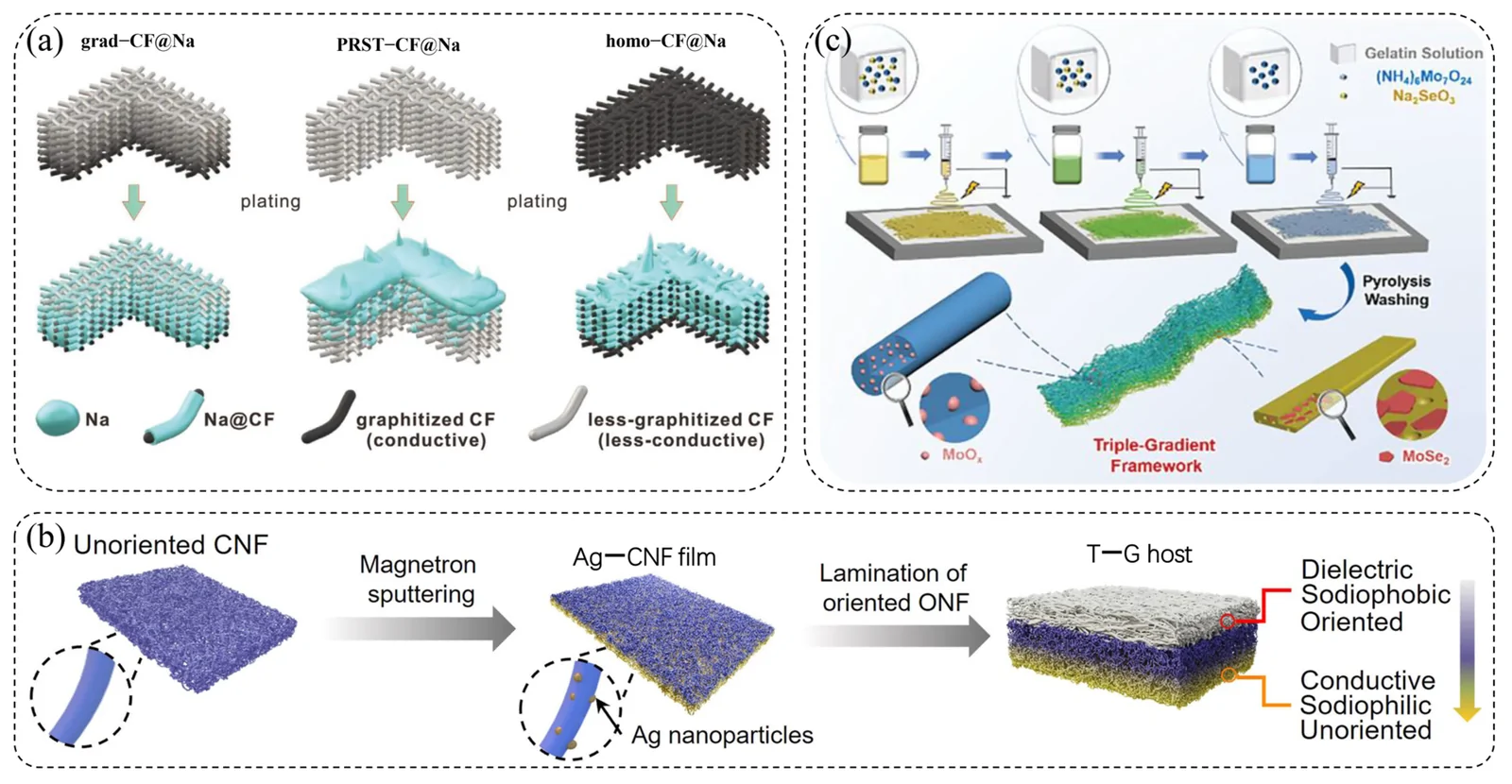
(**a**) Schematic diagram of Na plating in different collectors [89]. (**b**) Schematic of the preparation and main features of the T-G host [93]. (**c**) Schematic drawing of the synthesis process of Gra-GC-MoSe_2_ [94].

One of the core concepts of gradient design is to preferentially guide Na to deposit in the bottom region, which exhibits higher conductivity, by regulating the conductivity difference along the thickness direction of the current collector. Sun et al. designed a gradient graphitized 3D carbon fiber framework (grad-CF) as a host material [89]. In the design, as displayed in Figure 3a, the bottom layer consists of highly graphitized (highly conductive) carbon fibers, while the top layer is composed of lowly graphitized (low-conductivity) carbon fibers. When the low-conductivity side faces the separator, Na^+^ ions are more likely to gain electrons and undergo reduction in the highly conductive bottom region, facilitating “bottom-up” deposition away from the separator. Experimental results demonstrated that this gradient host could cycle stably at 6 mA cm^−2^, significantly mitigating the risk of dendrite penetration through the separator. Moreover, according to the previous discussion, the sodiophilic functional components can regulate the deposition behavior of Na^+^. Therefore, by constructing a gradient of sodiophilicity along the thickness direction of the current collector, the preferential nucleation sites for Na^+^ can be effectively regulated, guiding Na deposition to specific regions. Yue et al. developed a gradient sodiophilic carbon skeleton by adjusting the degree of disorder and defect concentration in carbon materials [79]. It was observed that metallic Na was preferentially deposited on the more disordered (and thus more sodiophilic) carbon spheres, gradually expanding into the carbon fiber regions, ultimately achieving dendrite-free Na deposition across the entire electrode. At 10 mA cm^−2^, the Na nucleation overpotential on this skeleton was merely 11.1 mV, and symmetric cells demonstrated stable cycling for 1000 h at 5 mA cm^−2^ and 5 mAh cm^−2^. Furthermore, He et al. constructed a stepped sodiophilic gradient 3D current collector using hydroxylated MXene (h-Ti_3_C_2_), with the h-Ti_3_C_2_ content increasing from top to bottom [90]. The highly sodiophilic h-Ti_3_C_2_ facilitated preferential Na^+^ deposition at the bottom of the current collector, enabling stable cycling for over 180 h, even under the challenging conditions of 40 mA cm^−2^ and 40 mAh cm^−2^. Moreover, the integration of multiple gradients allows for the more precise regulation of the Na deposition behavior [91,92]. For instance, Wu et al. designed a triple-gradient framework (denoted as T-G) that integrates gradient orientation, conductivity, and sodiophilicity (Figure 3b) [93]. The top layer consists of a highly oriented and electron-insulating oxidized polyacrylonitrile fibermat, while the bottom layer comprises randomly oriented, electron-conductive carbon nanofibers decorated with silver to enhance the sodiophilicity. Finite element simulations demonstrated that this triple-gradient scaffold optimized the distribution of the electric field and Na^+^ ions, effectively suppressing the “top-down” deposition mode. Even at high current densities and capacities of 5 mA cm^−2^ and 5 mAh cm^−2^, symmetric cells could cycle stably for over 1500 h. Similarly, as shown in Figure 3c, Cao et al. synthesized a self-supporting carbon framework with a triple-gradient structure (Gra-GC-MoSe_2_), which incorporated sodiophilic sites, the pore volume, and electrical conductivity, through a straightforward electrospinning–pyrolysis method [94]. The gradient distribution of selenium heteroatoms and MoSe_2_ and its derivatives tailored the sodiophilicity, while the pore volume gradient facilitated Na^+^ diffusion, and the conductivity gradient ensured that no “dead Na” was generated during the stripping process. This framework achieved a long cycle life of 2000 h in symmetric cells and operated stably even at a large capacity of 10 mAh cm^−2^.

It is important to recognize that the aforementioned properties—such as enhanced sodiophilicity, reduced nucleation overpotential, and high ionic conductivity—do not function independently but rather operate cooperatively to govern the overall deposition behavior. For instance, a current collector with exceptionally high sodiophilicity may promote uniform nucleation at the initial stage; however, if the ionic conductivity of the interface or the SEI formed thereon is insufficient, the subsequent growth of the Na deposit may still become non-uniform, leading to dendrite formation. Conversely, a highly conductive 3D scaffold with moderate sodiophilicity may achieve superior long-term stability by balancing nucleation promotion with sustained ion transport. Furthermore, the interfacial compatibility between the current collector and the electrolyte and the mechanical and chemical stability of the interface during repeated volume changes are equally critical factors that must be considered alongside sodiophilicity. Therefore, the design of an effective current collector should not rely on any single parameter but should instead pursue the synergistic optimization of multiple properties, including sodiophilicity, ionic/electronic conductivity, mechanical robustness, interfacial wettability, and structural integrity under cycling conditions. This holistic perspective is essential for achieving truly stable and dendrite-free SMAs.

### 3.2. Electrolyte Engineering

The electrolyte, as an indispensable component of the battery system, not only plays a crucial role in ion transfer and charge balance but also determines the energy density and electrochemical window of the battery system, as well as controlling the performance of the electrode/electrolyte interface [12,95]. As previously discussed, when the electrolyte comes into contact with Na metal, an SEI layer is spontaneously formed on its surface. This SEI layer significantly influences the interfacial characteristics, electrochemical stability, battery cycling efficiency, and suppression of Na dendrite growth. However, these properties are closely related to the composition of the SEI layer, meaning that the characteristics of the electrolyte dictate the performance of the SEI layer. Therefore, regulating the relevant components of the electrolyte plays a vital role in enhancing the performance of SMAs.

#### 3.2.1. Solvent and Salt Optimization

Conventional electrolytes predominantly consist of sodium salts and organic solvents, making the optimization of these components a fundamental strategy for enhancing the electrochemical performance of SMAs. The core principle involves regulating the solvation structure of the electrolyte—specifically, the coordination environment among Na^+^ ions, solvent molecules, and anions [96,97]. This regulation directs the preferential decomposition of either anions or solvent molecules on the electrode surface, thereby facilitating the formation of SEI layers with distinct compositions and characteristics. Extensive research indicates that the types and concentrations of solvents and salts significantly influence the cation coordination environment, which, in turn, determines the chemical composition, structural uniformity, and interfacial stability of the SEI [12,98]. These factors ultimately have a direct impact on the CE and cycle life of the battery.

The choice of solvent plays a decisive role in determining the Na^+^ solvation structure and the formation of the SEI. Conventional carbonate-based solvents, such as ethylene carbonate (EC), diethyl carbonate (DEC), and propylene carbonate (PC), exhibit strong electron-donating abilities that lead to strong coordination with Na^+^ ions. However, the decomposition products of these solvents typically result in a thick and non-uniform SEI that is rich in organic components and fails to effectively passivate the highly reactive Na metal surface [12,99]. For instance, when employing 1 M NaPF_6_ in carbonate-based electrolytes, Na||Cu cells often demonstrate CE below 25% and exhibit extremely poor cycling performance. In contrast, ether-based solvents, such as diglyme (G2) and tetraethylene glycol dimethyl ether (TEGDME), possess moderate solvation capabilities and lower reductive activity, which promote the formation of a thinner and denser SEI enriched in inorganic components, such as NaF and Na_2_O [100,101,102]. Recently, Seh et al. demonstrated that utilizing 1 M NaPF_6_ in a G2 electrolyte enabled Na||Cu cells to achieve CE as high as 99.9% after 300 cycles, with the deposition morphology exhibiting dense, dendrite-free granular structures [103]. Furthermore, Le et al. systematically compared the performance of various sodium salts in TEGDME solvent and found that 1 M NaBF_4_ in TEGDME facilitated over 1000 stable cycles with CE of 99.9%, attributed to the formation of a uniform SEI dominated by inorganic components [104].

The type of sodium salt is crucial in determining the solvation structure and SEI properties, as different anions exhibit distinct coordination abilities, reductive stability, and decomposition products that significantly affect battery performance. In the same G2 solvent, Seh et al. systematically compared multiple sodium salts, including NaPF_6_, NaTFSI, NaFSI, NaOTf, and NaClO_4_, and found that NaPF_6_ exhibited the best performance, achieving high CE of 99.9% over 300 cycles, while NaClO_4_ demonstrated the poorest performance, with rapid CE decay during cycling [103]. This difference can be attributed to the varying capabilities of different anions to participate in the Na^+^ solvation sheath and the properties of their decomposition products: PF_6_^−^ can effectively decompose to form NaF, which possesses high ionic conductivity and excellent mechanical strength, whereas the decomposition products of ClO_4_^−^ lack effective passivation capabilities for sodium metal. Furthermore, the salt concentration plays a significant role in regulating the solvation structure [105,106]. In highly concentrated electrolytes (HCEs), the substantial reduction in free solvent molecules forces anions into the primary solvation sheath of Na^+^, forming solvation structures dominated by contact ion pairs (CIPs) and aggregates (AGGs) [97]. This anion-rich coordination environment promotes the preferential reduction and decomposition of anions on the electrode surface, resulting in the formation of a stable, inorganic-rich SEI. Lee et al. reported that an ultra-concentrated 5 M NaFSI/DME electrolyte, leveraging this principle, achieved high CE of 99.3% after 120 cycles in Na||stainless steel cells while effectively suppressing an aluminum current collector’s corrosion at high voltages [107]. However, HCEs typically suffer from high viscosity, low ionic conductivity, and high costs, which limit their large-scale application [108].

**Figure 4:**
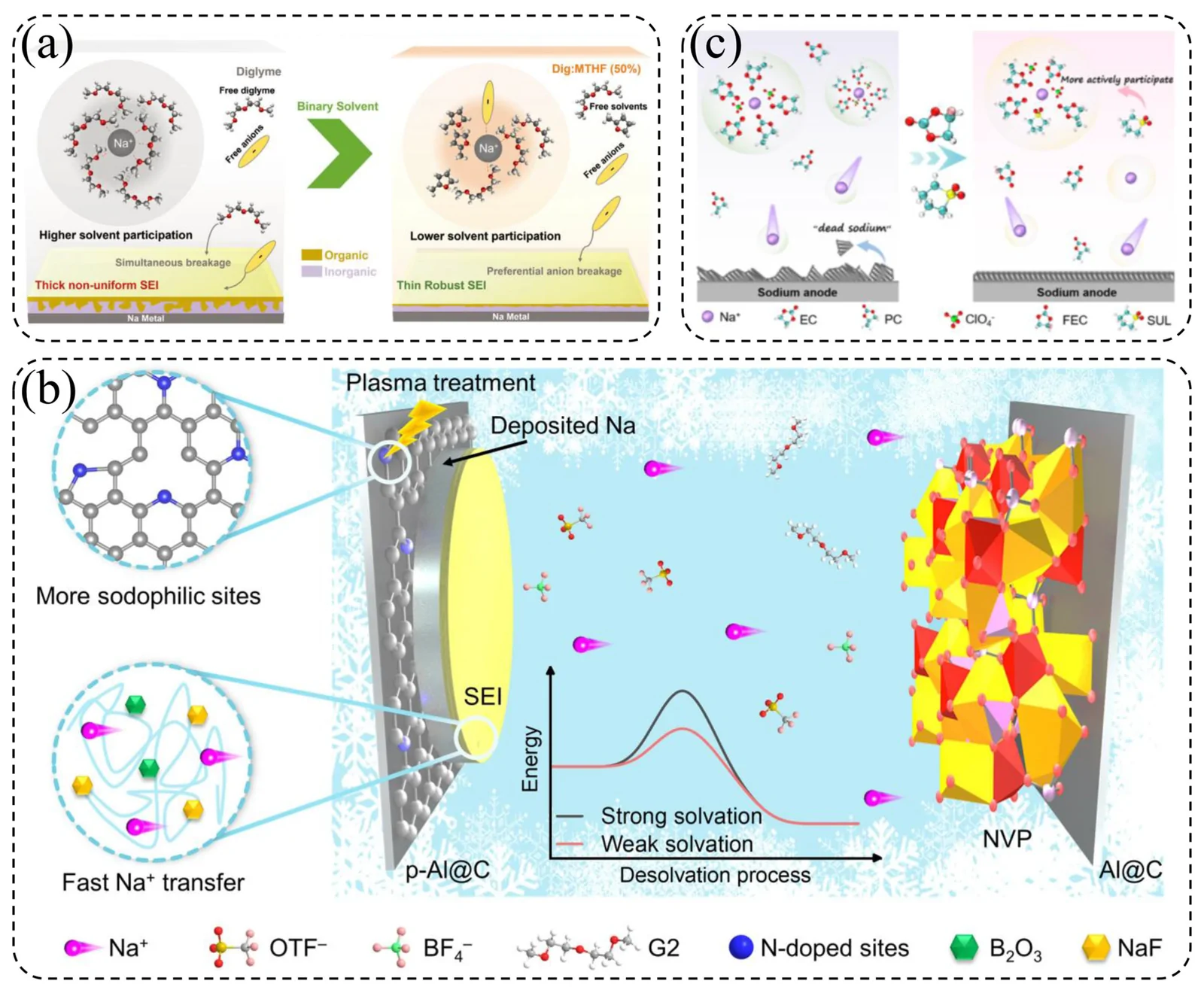
(**a**) Schematic illustration of SEI formation impacted by the solvation structure in different electrolyte systems [113]. (**b**) Illustration of the desolvation behavior of the NaOTF-NaBF_4_-G2 dual-salt system and a conventional electrolyte [115]. (**c**) Schematic diagram of the mechanism behind the dual-function roles of FEC and SUL in SEI formation [121].

To circumvent the inherent drawbacks of HCEs, researchers have proposed strategies that utilize co-solvents or co-salts to regulate the solvation structure, thereby achieving anion enrichment effects comparable to those of HCEs at conventional salt concentrations [109,110,111]. The co-solvent strategy focuses on the introduction of a secondary solvent with a weak solvation ability. The differential interactions among the co-solvent, primary solvent, and Na^+^ effectively modulate the coordination environment. For instance, Zhang et al. introduced tetraethylene glycol dimethyl ether (G4) as a co-solvent in 1 M NaPF_6_ within a G2 electrolyte, finding that the addition of G4 resulted in two new solvation structures: Na-(G2)-(G4) and Na-(G4)-PF_6_ [112]. This modification weakened the coordination strength between Na^+^ and PF_6_^−^ as well as G2, facilitating the greater entry of PF_6_^−^ into the primary solvation sheath. Consequently, this led to the formation of an inorganic-rich SEI on the current collector, significantly enhancing the uniformity and reversibility of Na metal deposition. Moreover, Vaidyula et al. introduced 2-methyltetrahydrofuran (MTHF) as a co-solvent in 1 M NaPF_6_ within a G2 electrolyte [113]. Through DFT calculations and molecular dynamics (MD) simulations, they demonstrated that MTHF, due to its weaker electron-donating ability and greater steric hindrance, preferentially interacted with PF_6_^−^ anions rather than Na^+^. This interaction promotes the formation of a solvation structure dominated by CIPs. As shown in Figure 4a, the CIP-dominated coordination environment subsequently decomposed on the electrode surface, forming a uniform SEI characterized by an outer organic layer and an inner inorganic layer, which effectively suppressed Na dendrite growth. Based on this strategy, Na||Na symmetric cells utilizing G2:MTHF (50%, *v*/*v*) achieved a cycle life exceeding 7000 h with CE of 99.72%.

In contrast to the co-solvent strategy, the co-salt strategy involves the introduction of a second cation into the electrolyte. This approach exploits the differential coordination abilities of various cations with solvents and anions to construct a “hybrid cation solvation structure”, facilitating the synergistic optimization of the interfacial chemistry. Li et al. investigated the performance of an electrolyte comprising 0.9 M NaPF_6_ and 0.1 M NaBF_4_ in G2 [114]. MD simulations revealed that both PF_6_^−^ and BF_4_^−^ could enter the primary solvation layer of Na^+^, with Na^+^ displaying a preference for coordinating with BF_4_^−^, thereby effectively modulating the composition of the solvation environment. Zhu et al. further examined the changes in solvation structure within the NaOTF-NaBF_4_-G2 dual-salt system, discovering that the introduction of OTF^−^ and BF_4_^−^ significantly weakened the interaction between Na^+^ and G2 (Figure 4b) [115]. The coordination number between Na^+^ and the oxygen atoms of G2 decreased from 5.85 to 5.38, while the electrostatic potential charges of OTF^−^ and BF_4_^−^ were lower than that of PF_6_^−^. This resulted in longer Na^+^-O(G2) bond lengths and a diminished solvation effect in the dual-salt system. More critically, NaBF_4_, due to its higher lowest unoccupied molecular orbital (LUMO) energy level, decomposes preferentially before NaOTF, leading to the formation of boron-rich inorganic species in the SEI. These inorganic components, characterized by high mechanical strength and favorable ionic conductivity, significantly enhanced the chemical and mechanical stability of the SEI. The advantages of this dual-salt strategy were clearly demonstrated in the electrochemical performance. The work by Zhang et al. further extends this concept by introducing Li^+^ salts into Na-based electrolytes [112]. This approach utilizes the competitive coordination effect between Li^+^ and Na^+^ to shift the Na^+^ solvation structure toward an anion-rich configuration, thereby forming an inorganic SEI that is rich in both LiF and NaF. This hybrid cation strategy demonstrated significant advantages in enhancing the interfacial stability and reducing desolvation energy barriers.

#### 3.2.2. Additives

Additive engineering is one of the most attractive strategies for stabilizing SMAs because of its simplicity, cost-effectiveness, and remarkable efficacy. The introduction of a small amount of functional additives can significantly modulate the Na^+^ solvation structure, alter the composition and properties of the SEI, or regulate the Na deposition behavior via electrostatic shielding, thereby establishing a stable interfacial chemistry and enabling uniform Na deposition [116,117]. Based on their primary mechanisms of action, additives can be classified into three categories: film-forming molecular additives, ionic additives for electrostatic shielding, and multifunctional synergistic systems.

Film-forming molecular additives primarily function by preferentially decomposing to participate in SEI construction or by regulating the Na^+^ solvation structure. Fluoroethylene carbonate (FEC) is among the most extensively studied anionic additives. Due to its low LUMO energy level, FEC undergoes preferential reductive decomposition on the sodium surface, forming a NaF-rich inorganic SEI that effectively suppresses dendrite growth and enhances the cycling stability [118,119]. However, Wang and Hofmann systematically compared FEC with sodium difluoro(oxalato)borate (NaDFOB) in a 1 M NaPF_6_ EC/PC electrolyte [120]. They found that the addition of only 1–2 wt% NaDFOB extended the cycle life of Na||NVP cells from less than 75 cycles to 600 cycles, with capacity retention of approximately 96%. In contrast, while FEC is beneficial for half-cell performance, it exhibits poor compatibility with hard carbon anodes in full cells. This indicates that the effects of additives on anode and cathode interfaces differ significantly, and the choice of additive must consider its compatibility with the entire battery system. Additionally, sulfolane (SUL) additives, characterized by highly polar sulfone groups, can effectively tailor the Na^+^ solvation structure, mitigate excessive anion decomposition under high-voltage conditions, and form sulfur-containing cathode electrolyte interphase (CEI) species, thereby enhancing the oxidative stability of the electrolyte [121].

Ionic additives regulate Na deposition primarily through the electrostatic shielding effect. When Na dendrite tips form, the introduced cations (e.g., K^+^, Li^+^), which possess lower reduction potentials than Na^+^, are not reduced but preferentially adsorb onto the dendrite tips. This process results in the formation of a positively charged shielding layer that repels incoming Na^+^ ions, thereby forcing deposition in adjacent regions. Shi et al. first introduced potassium bis(trifluoromethylsulfonyl)imide (KTFSI) as a bifunctional additive [122]. The TFSI^−^ anion decomposes to form an SEI containing Na_3_N and NaN_x_O_y_, while the K^+^ cation provides electrostatic shielding. This synergistic effect enabled Na||Na symmetric cells to achieve an ultralong cycle life of 2700 h at 1 mA cm^−2^ and 1 mAh cm^−2^ and even 480 h under harsh conditions of 2 mA cm^−2^ and 10 mAh cm^−2^. Similarly, Zhang et al. introduced LiBF_4_ into ether-based electrolytes, where the electrostatic shielding effect of Li^+^ and the NaF formed by BF_4_^−^ decomposition worked in concert, allowing Na||Na symmetric cells to cycle stably for 3000 h at 1 mA cm^−2^ [123]. Moreover, Chen et al. further demonstrated that the addition of Li^+^ salts to NaOTf/DME electrolytes altered the solvation environment, as Li^+^ exhibits a higher binding energy with DME than Na^+^, thereby stabilizing the electrolyte and suppressing Na dendrite growth [124]. Furthermore, anionic additives can also play a crucial role. For instance, sulfur-containing additives like Na_2_S_6_ demonstrate unique advantages. Wang et al. found that Na_2_S_6_ in ether-based electrolytes constructed a stable SEI that was rich in Na_2_O, Na_2_S_2_, and Na_2_S, enabling stable Na plating/stripping for over 100 cycles at a high current density of 10 mA cm^−2^ [125].

Multifunctional and dual-additive synergistic systems integrate multiple functions to simultaneously regulate both the anode and cathode interfaces. Yang et al. proposed a dual-additive system that combined FEC and a sulfur-containing additive (SUL) (Figure 4c) [121]. In this system, FEC preferentially decomposes on the anode side, forming a dense NaF-rich SEI, while SUL modulates the Na^+^ solvation structure on the cathode side and constructs a sulfur-containing cathode electrolyte interphase (CEI). This bidirectional interfacial regulation enabled Na||Na symmetric cells to cycle for 1400 h at 0.5 mA cm^−2^ and 0.5 mAh cm^−2^, while Na||NVP full cells retained over 88% capacity after 1100 cycles at a current density of 80 mA g^−1^. Wang et al. employed a dual-additive approach in an all-fluorinated electrolyte using sodium difluoro(oxalato)borate (NaDFOB) and potassium 2-thienyltrifluoroborate (KTFPB) [126]. They found that NaDFOB promoted the decomposition of KTFPB at the Na interface, resulting in the formation of a Na_2_S-rich, highly conductive SEI. Additionally, the decomposition products of both additives collaboratively constructed a boron–oxygen (B–O)-rich CEI that enhanced the mechanical integrity. This synergistic effect increased the average CE of Na||Cu cells to 99.4% and enabled Na||NVP full cells with a low sodium-to-active-material ratio of 2:1 to achieve 200 stable cycles. These studies underscore that dual-additive strategies, through synergistic cooperation among components, can effectively address multiple challenges such as dendrite growth and interfacial instability, presenting a promising avenue for the electrolyte design of high-performance SMBs.

### 3.3. Artificial SEI Engineering

The SEI layer plays a crucial role in SMAs, as it not only isolates Na metal from the electrolyte but also regulates the deposition behavior of Na^+^ and suppresses the growth of Na dendrites [32]. Therefore, a stable SEI layer is essential for achieving a long cycle life in SMAs. An ideal SEI typically possesses characteristics such as excellent electrical insulation, high ionic conductivity, substantial mechanical strength, and uniform electric field distribution [12]. However, it is essential to emphasize that these properties must function synergistically rather than in isolation. A high Young’s modulus, for instance, is often cited as a key factor for mechanically suppressing dendrite penetration; yet, if the SEI lacks sufficient electronic insulation, continuous side reactions between the Na metal and the electrolyte will persist, leading to sustained SEI growth and eventual capacity fade. Similarly, an SEI with high ionic conductivity but poor mechanical flexibility may crack upon volume expansion, exposing fresh Na surfaces and triggering new dendrite nucleation. Furthermore, the thickness of the SEI layer presents a critical trade-off: an overly thick SEI may offer better mechanical protection but at the cost of increased ionic resistance and reduced overall energy density, whereas an excessively thin SEI may fail to provide adequate passivation. Beyond these factors, the structural evolution of the SEI during prolonged cycling—including its tendency to crack, delaminate, or undergo phase transformation—must also be carefully considered. Therefore, the rational design of artificial SEI layers demands comprehensive, multiparameter optimization that balances ionic conductivity, electronic insulation, mechanical strength, flexibility, thickness control, and long-term structural stability. Moreover, the SEI that naturally forms upon contact between the Na metal and the electrolyte is usually loose and discontinuous, leading to uneven ionic diffusion and consequently causing issues such as the formation of Na dendrites [32,43]. To address these challenges, constructing a robust artificial SEI interface layer through pretreatment methods represents a feasible strategy [127]. Based on the composition, SEI layers can be classified into inorganic and inorganic–organic hybrid interface layers.

#### 3.3.1. Artificial Inorganic SEIs

Inorganic materials have become a core choice for the design of artificial SEIs due to their high ionic conductivity, high Young’s modulus, excellent corrosion resistance, and structural stability [128,129]. Among the various inorganic components, NaF is recognized as a key component for constructing stable SEIs due to its outstanding electronic insulation properties, high interfacial energy, and low diffusion energy barrier for Na^+^ [130,131]. Researchers have developed various chemical pretreatment methods to construct NaF-rich artificial SEIs on Na surfaces [132,133]. Cheng et al. utilized the spontaneous reaction between Na and triethylamine trifluoride to build a NaF-rich artificial SEI on the Na surface. This structure not only exhibited excellent ductility to alleviate the stress generated during the plating/stripping processes but also maintained structural integrity during cycling, allowing the modified Na electrode to stably cycle for over 1000 h [134]. Furthermore, the strategy of constructing a controllable NaF-rich artificial interfacial layer through the reaction of perfluoropolyether with Na also demonstrated an outstanding electronic barrier and cycling stability [135]. Inspired by the NaF artificial interfacial layer, Tian et al. synthesized a NaI-rich artificial interfacial layer through the reaction of Na with 1-iodopropane solution. Symmetric batteries modified with the NaI artificial interfacial layer also exhibited good cycling performance [136]. Furthermore, Archer’s team employed theoretical methods to calculate the diffusion barriers of Na^+^ in various NaX (X = F, Cl, Br, I) phases [137]. The theoretical calculations indicated that NaBr possessed the lowest Na^+^ diffusion barrier, meaning that it can significantly facilitate the rapid diffusion and uniform deposition of Na^+^. Based on this theoretical result, they treated metallic Na with a bromopropane solution to obtain a type of SMA with a NaBr protective layer. SEM images and in situ optical images confirmed that the artificial NaBr layer not only alleviated the formation of dendrites but also effectively prevented side reactions between the Na electrode and the electrolyte, resulting in an improved cycling life in symmetric batteries.

While constructing an artificial SEI on the surface of a Na electrode is an effective strategy to enhance the performance of SMAs, the mechanical toughness of the SEI is often underestimated. It is widely accepted that structurally unstable and brittle SEIs are inadequate in mitigating volume changes and suppressing dendrite growth. Therefore, it is essential to rationally design and fabricate artificial SEIs that possess both high ionic conductivity and excellent mechanical toughness for SMAs. Recently, Yu’s team constructed a Na_3_P artificial SEI layer on the Na surface through the in situ reaction of highly reactive Na metal with red phosphorus [138]. This artificial inorganic layer not only exhibited ionic conductivity of up to 0.12 mS cm^−1^ but also had a Young’s modulus of 8.6 GPa. Furthermore, they provided further evidence through DFT calculations and experimental results that the prepared Na_3_P inorganic SEI possessed relatively low Na^+^ diffusion energy. These characteristics facilitate the rapid diffusion transport of Na^+^ within the SEI, enabling uniform Na deposition, while its high Young’s modulus effectively prevents dendrite growth. Consequently, symmetric cells assembled with Na electrodes modified with this Na_3_P artificial SEI layer could stably cycle for over 780 h under conditions of 1 mA cm^−2^ and 1 mAh cm^−2^. Similarly, Yang et al. utilized ab initio molecular dynamics (AIMD) calculations to examine Na^+^ migration in the phases of Na_2_O, Na_2_S, Na_2_Se, and Na_2_Te and revealed that Na_2_Te possessed a low Na^+^ migration energy barrier and a high diffusion coefficient of 2.4 × 10^−3^ cm^2^ s^−1^. This indicates that Na_2_Te facilitates the rapid transport of Na^+^. Furthermore, DFT calculations demonstrated that Na_2_Te exhibited high adsorption energy for Na^+^, which promotes the swift adsorption of Na^+^ on its surface and enables uniform Na deposition due to the high ionic conductivity of Na_2_Te. Additionally, experimental tests indicated that the artificially created Na_2_Te layer possessed a Young’s modulus as high as 8.7 GPa, providing favorable conditions to suppress dendrite growth and consequently extending the electrode’s plating/stripping performance. Based on these exceptional characteristics, the symmetric battery assembled with Na electrodes protected by the Na_2_Te artificial SEI layer could stably cycle for 700 h at 1 mA cm^−2^ and 1 mAh cm^−2^ [139].

**Figure 5:**
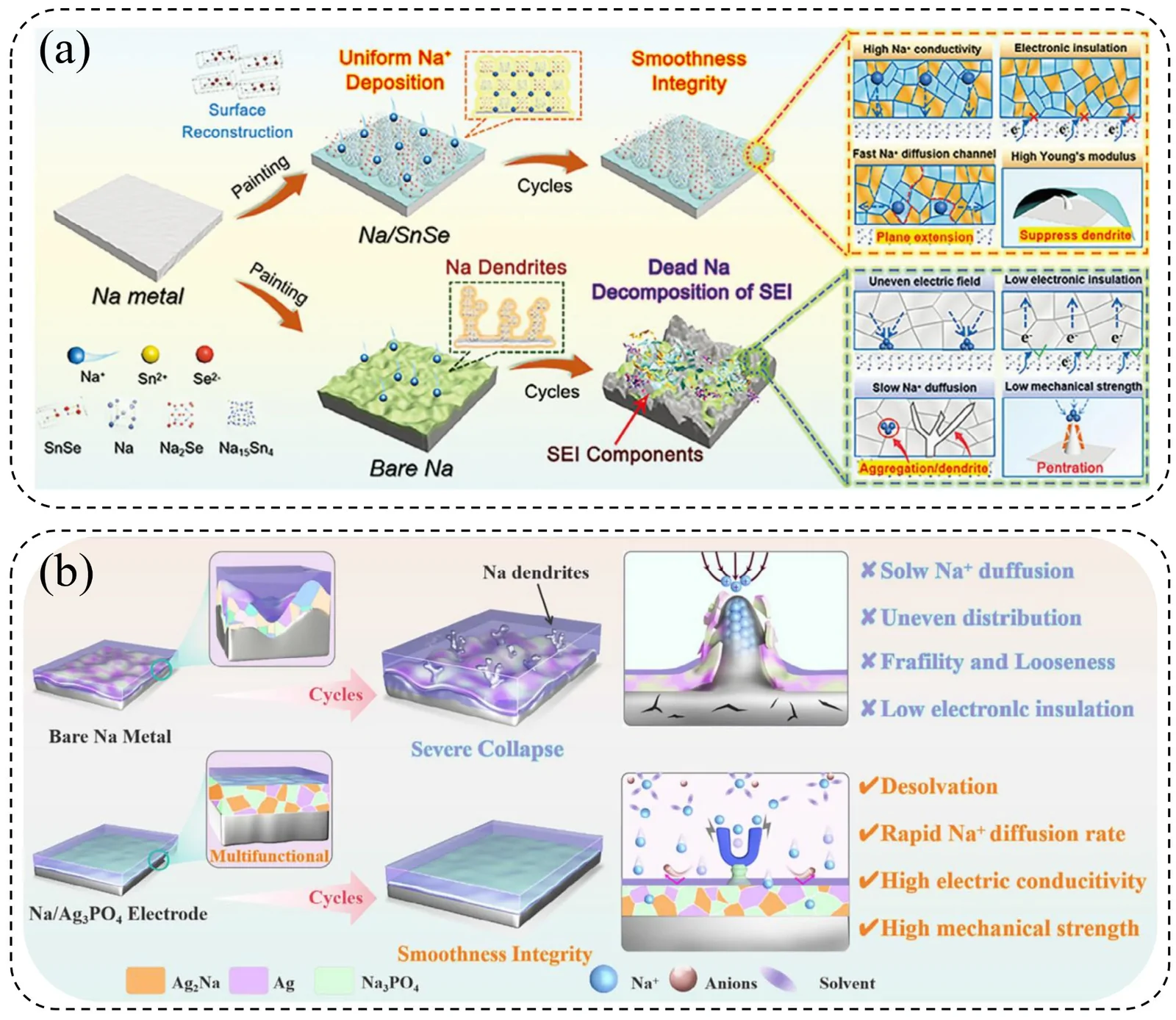
(**a**) Illustration of the synthesis process of an artificial SEI layer and the evolutionary mechanism [141]. (**b**) Illustration of the evolutionary mechanism of a Ag_2_Na/Ag/Na_3_PO_4_ triphasic artificial SEI [143].

**Figure 6:**
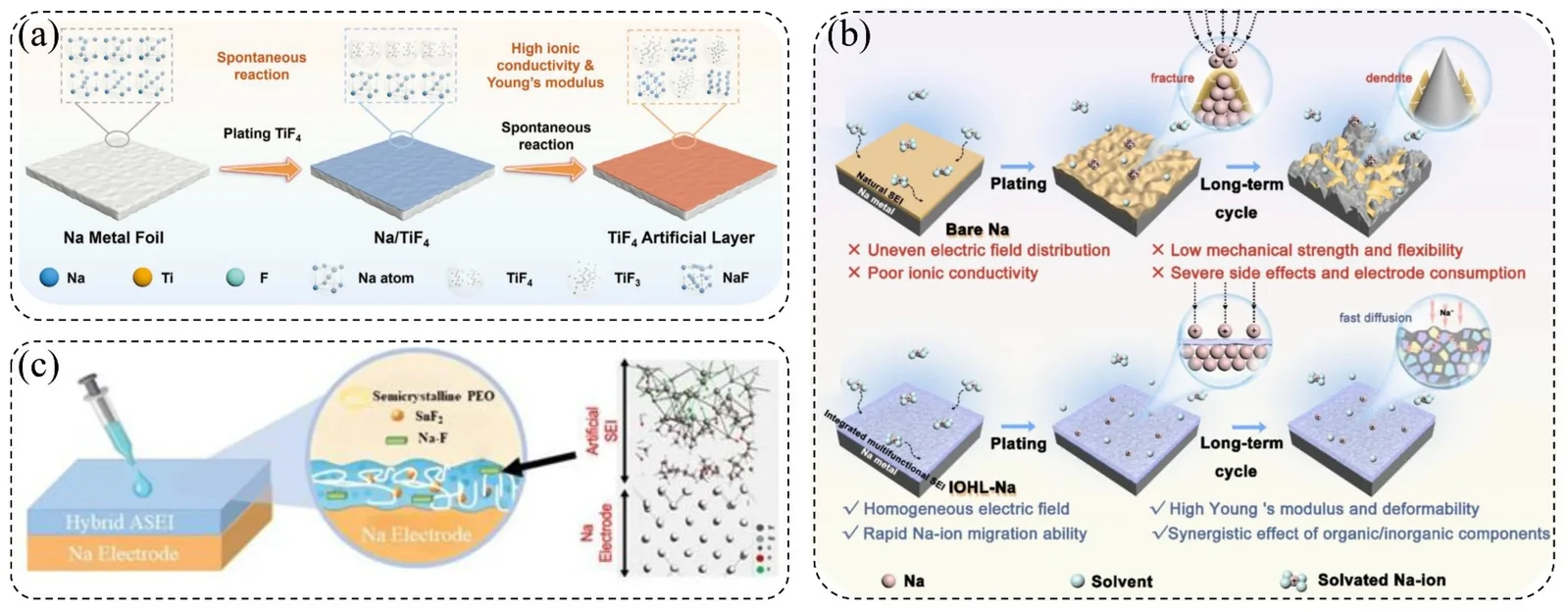
(**a**) Schematic drawing of the fabrication process of a NaF/TiF_3_ heterointerface [145]. (**b**) Schematic image showing a uniform inorganic–organic hybrid layer and its evolution process with different functional characteristics [153]. (**c**) Illustration of the synthesis process of an artificial hybrid interface [154].

With the advancement of research, it has become evident that a single component is insufficient to meet all the requirements of an ideal SEI. Consequently, the construction of multicomponent artificial SEIs, composed of various inorganic phases, has emerged as a prominent research focus. The fundamental concept revolves around leveraging the synergistic effects of different components to attain functional complementarity. Under this design paradigm, researchers have developed a variety of high-performance multicomponent artificial SEI systems [128,129,140]. For instance, Cao et al. constructed a Na_15_Sn_4_/Na_2_Se heterointerface through the in situ reaction of SnSe with Na (Figure 5a) [141]. The highly sodiophilic Na_15_Sn_4_ alloy enhanced interfacial bonding and suppressed structural failure caused by volume changes, while the Na_2_Se superionic conductor optimized the Na^+^ conduction efficiency. Their synergy enabled symmetric cells to cycle for over 2400 h. Recently, our group created a Na_2_Se/V interphase by reacting VSe_2_ with Na, leveraging the high sodiophilicity of V and the excellent ionic conductivity of Na_2_Se to achieve 1790 h of stable cycling in carbonate-based electrolytes [142]. Similarly, Qi et al. created a Ag_2_Na/Ag/Na_3_PO_4_ triphasic artificial SEI through the reaction of Ag_3_PO_4_ with Na (Figure 5b), where the Ag_2_Na and Ag components ensured good interfacial electronic conduction, and Na_3_PO_4_ modulated the interfacial ion flux through its desolvation capabilities, enabling symmetric cells to cycle stably for over 1600 h [143]. Moreover, building upon the prominent characteristics of NaF discussed previously, numerous researchers have integrated it into multicomponent functional SEI systems to enhance the performance of SMAs. For example, Li et al. developed a Na_3_Bi/NaF heterointerface through the reaction of Na with BiF_3_, where abundant grain boundaries and built-in electric field effects significantly accelerated interfacial charge transfer and ion diffusion, resulting in a cycle life exceeding 2000 h at 2 mA cm^−2^ [144]. Moreover, Guo et al. constructed a NaF/TiF_3_ heterointerface via the in situ reaction of TiF_4_ with Na (Figure 6a), where the TiF_3_ phase provided a strong Na^+^ binding energy and excellent sodiophilicity, while the NaF phase offered fast ion diffusion pathways [145]. Their synergy enabled symmetric cells to achieve stable cycling for 2370 h at 0.5 mA cm^−2^.

In addition to component design, precise control of parameters such as the thickness, uniformity, and interfacial affinity of artificial SEIs is also crucial. Luo et al. successfully constructed a mixed SEI layer of Na_3_P-NaBr on the surface of Na by immersing Na strips in a DME solution containing PBr_3_, combined with a rolling process [146]. This method allows for precise control of the SEI thickness by adjusting the concentration of PBr_3_. Theoretical calculations indicate that the Na_3_P phase provides a rapid bulk diffusion pathway for Na^+^, while the NaBr phase ensures the rapid migration of Na^+^ along the interface due to its low surface diffusion energy barrier and large band gap. Together, these two phases enhanced the interfacial stability and the uniform distribution of ions, enabling the modified sodium electrode to achieve a long lifespan with stable cycling for over 700 h. Moreover, Shi et al. used an in situ chemical reaction between metallic Na and a solution of Na acetate and propylene carbonate, and a thickness-controllable bifunctional protective layer composed of Na acetate and Na antimonide (SA) was prepared on the surface of a Na substrate [147]. The two components, Na antimonide and Na acetate, form a synergistic effect; the former reduces the contact angle between the electrolyte and the electrode, achieving uniform wetting across the entire area, while the latter relies on strong adsorption energy to enrich Na^+^ at the interface, thereby reducing the deposition overpotential. Together, these components mitigate the differences in ionic transport at the interface, resulting in smooth, dendrite-free Na deposition. Additionally, the composite interface exhibited a Young’s modulus of 6.7 GPa, enabling it to withstand volume stress during charge and discharge cycles, while also promoting the decomposition of fluoroethylene carbonate in the electrolyte, generating high-mechanical-strength NaF to reinforce the interface. Owing to these advantages, the SA-modified symmetric battery could maintain stable cycling for 2166 h under ambient conditions.

#### 3.3.2. Artificial Inorganic–Organic Hybrid SEIs

While inorganic artificial SEI layers offer advantages in ionic conductivity and mechanical strength, their inherent brittleness often renders them inadequate in accommodating the substantial volume fluctuations of Na metal during prolonged cycling. Conversely, organic SEI components, while flexible, typically lack the requisite mechanical robustness to effectively suppress dendrite penetration. To synergistically integrate the complementary properties of both material classes, researchers have developed artificial inorganic–organic hybrid SEI layers. These hybrid structures aim to combine the chemical stability and ionic conductivity of inorganic species with the mechanical flexibility of organic polymers, thereby establishing a more durable and adaptive protective interphase for SMAs [148,149].

Atomic layer deposition (ALD) and molecular layer deposition (MLD) techniques provide unique advantages for the fabrication of hybrid layers, allowing for precise control over the thickness and composition at the molecular level under mild conditions [95]. For instance, Zhao et al. employed MLD to synthesize an inorganic–organic alucone layer on Na metal using trimethylaluminum and ethylene glycol as precursors [150]. Theoretical calculations revealed that Na-O and Na-Al bonds significantly enhanced interfacial interactions, thereby promoting uniform sodium deposition. By systematically varying the layer architecture, they demonstrated that Na anodes modified with a 25-cycle alucone coating achieved the longest cycling life of 270 h with minimal voltage fluctuations. Extending this approach, Pirayesh et al. utilized a combined ALD/MLD process to precisely engineer the structure, thickness, and composition of hybrid interfaces, achieving a transition from nanolaminate to nanoalloy structures [151]. Their results indicated that the nanoalloy interface exhibited the most stable electrochemical performance. Employing a combination zone model, they correlated the mechanical stability of different interfaces with electrochemical performance; the optimized hybrid interface enabled symmetric cells to operate for over 2000 h at 1 mA cm^−2^ and 1 mAh cm^−2^. These studies underscore the efficacy of ALD/MLD in tailoring hybrid interfaces for enhanced SMAs.

Despite the precision of ALD and MLD, their scalability and widespread application remain constrained by high costs and the need for specialized equipment. In response, more accessible strategies that leverage the high reactivity of Na metal have been explored for the in situ construction of inorganic–organic hybrid SEIs. For instance, Yu’s group utilized an in situ reaction between poly(vinylidene fluoride) powder and Na metal to fabricate a composite SEI layer (IOHL) that was rich in inorganic NaF and organic components with abundant carbon–carbon (C-C) and carbon–fluorine (C-F) bonds [152]. The resulting IOHL exhibited a high Young’s modulus of 10.6 GPa, effectively enhancing the mechanical toughness, accommodating volume expansion, and suppressing dendrite growth. Theoretical simulations confirmed strong Na^+^ adsorption on the IOHL and its ability to regulate Na^+^ distribution, thereby enabling uniform deposition. Consequently, IOHL-modified symmetric cells achieved stable cycling for over 770 h at 1 mA cm^−2^ and 1 mAh cm^−2^. Similarly, Zhu et al. designed a multiphase artificial SEI through the in situ reaction of sulfur (S_8_) and para-dichlorobenzene with Na, forming a hybrid layer composed of PhS_2_Na_2_, Na_2_S, and NaCl [148]. DFT calculations revealed that PhS_2_Na_2_ exhibited a weak Na^+^ binding energy, promoting Na^+^ migration, while the inorganic components synergistically facilitated Na^+^ diffusion, enabling stable cycling for over 800 h at 1 mA cm^−2^. Similarly, Jiao’s group further developed a robust, flexible, and uniform inorganic–organic hybrid layer that was approximately 6 μm thick by directly reacting Na with a mixed solution of tin (II) chloride (SnCl_2_) and 4-chloro-2,6-dimethylphenol [153]. As illustrated in Figure 6b, this layer consisted of polyphenylene oxide, sodium chloride (NaCl), and nanocrystalline tin (nano-Sn). The uniformly dispersed nano-Sn and NaCl within the organic matrix ensured adequate mechanical integrity and facilitated rapid Na^+^ diffusion channels. Furthermore, the self-polymerized organic component inhibited the agglomeration of inorganic particles, thereby maintaining a homogeneous electric field and accommodating volume expansion. This rational design allowed symmetric cells to sustain a prolonged cycle life exceeding 2000 h even at 4 mA cm^−2^. In a notable advancement of the hybrid SEI concept, Damircheli et al. recently reported a synergistic organic–inorganic artificial SEI formed through the spontaneous conversion of tin fluoride (SnF_2_) within a poly(ethylene oxide) (PEO) matrix [154]. As shown in the schematic illustration in Figure 6c, via drop-casting a PEO:SnF_2_ solution onto Na metal, they created a hybrid layer that underwent in situ chemical conversion upon contact with Na, generating NaF-rich domains and a Na-Sn alloy network uniformly distributed throughout the interphase. This dynamic, self-reinforcing structure synergistically combines the flexibility of the PEO matrix with the mechanical robustness and thermodynamic stability of the inorganic conversion products. The molecular dynamics simulations confirmed the spontaneous formation of NaF clusters and a Na-Sn alloy network that homogenized Na^+^ flux and fortified the hybrid layer. Optimized coatings dramatically extended the symmetric cell cycle life from approximately 100 h for bare Na electrodes to nearly 760 h at 0.5 mA cm^−2^ in carbonate electrolytes. Moreover, the full cells paired with NVP cathodes delivered a stable capacity of approximately 112–122 mAh g^−1^ over 100 cycles, with approximately 99% CE. This work highlights a potential paradigm for designing dynamic, self-adaptive hybrid interfaces through spontaneous inorganic conversion within a polymer matrix, offering a new avenue for achieving ultrastable artificial inorganic–organic hybrid SEIs.

To facilitate a more direct and meaningful comparison among the various strategies discussed above, Table 1 provides a summary of representative examples from each category: current collector engineering, electrolyte engineering, and artificial SEI engineering. This table highlights their key performance parameters, primary advantages, and remaining limitations.

## 4. Conclusions and Perspectives

In summary, SMAs are considered the most promising anode materials for next-generation high-energy-density SMBs due to their ultrahigh theoretical specific capacity of 1166 mAh g^−1^ and low electrochemical potential of −2.71 V versus the standard hydrogen electrode. However, their practical application is significantly impeded by a series of interrelated challenges, including the instability of SEI films, which undergo continuous dynamic evolution, severe volume fluctuations, and structural failures due to their hostless nature. Additionally, uncontrollable dendrite growth poses safety hazards, leading to low CE and a limited cycle life. The root causes of these issues lie in the intrinsically high reactivity of Na metal, the absence of a physical support framework during deposition, and the uneven distribution of the ionic flux and electric fields. Together, these factors constitute the core bottlenecks that restrict the development of SMBs. To address these challenges, extensive strategies have been developed to stabilize SMAs. This review systematically summarizes three major categories: current collector engineering, electrolyte engineering, and artificial SEI engineering. Current collector engineering involves the design of planar, 3D, and gradient architectures to regulate the local current density and Na^+^ flux, guiding uniform nucleation and enabling “bottom-up” dendrite-free deposition. Electrolyte engineering focuses on optimizing solvents, salts, and functional additives to tailor the solvation structure, thereby constructing robust inorganic-rich SEI layers and utilizing electrostatic shielding effects to suppress dendrite formation. Meanwhile, artificial SEI engineering aims to pre-construct inorganic or inorganic–organic hybrid protective layers, establishing a physicochemical barrier between the electrode and electrolyte that combines high ionic conductivity, superior mechanical strength, and sufficient flexibility. Significant progress has been made in all these areas, with some systems achieving stable cycling over thousands of hours, demonstrating the feasibility of overcoming the intrinsic drawbacks of SMAs through rational design. Nevertheless, it is crucial to recognize that the successful stabilization of SMAs does not arise from optimizing any single parameter, such as sodiophilicity, ionic conductivity, or mechanical strength, in isolation. Rather, it results from the careful and synergistic integration of multiple functionalities that collectively address the complex interplay among nucleation, ion transport, interfacial chemistry, and mechanical integrity. Future design efforts must adopt a holistic perspective that simultaneously considers interfacial compatibility, electronic insulation, SEI thickness control, and the dynamic structural evolution of the electrode/electrolyte interface during prolonged cycling, rather than pursuing individual performance metrics independently.

Importantly, a critical caveat must be acknowledged when interpreting the impressive cycling data reported in the literature: the vast majority of these results, including those summarized in this review, were obtained under idealized laboratory conditions that differ substantially from practical industrial requirements. Most long-term cycling tests are conducted in symmetric cells or half-cells at relatively low current densities (typically < 5 mA cm^−2^) and low areal capacities (typically < 5 mAh cm^−2^), with abundant sodium metal (high N/P ratio) and excess electrolyte. In stark contrast, commercially relevant conditions demand high areal capacities (>5 mAh cm^−2^), high current densities (>10 mA cm^−2^), lean electrolyte conditions (low electrolyte-to-capacity ratio), limited sodium excess (low N/P ratio, ideally approaching anode-free configurations), and stable full-cell operation with high-loading cathodes. These practical requirements impose significantly more stringent demands on the interfacial stability, ion transport kinetics, and mechanical integrity of the anode. Therefore, the research community should place greater emphasis on validating promising strategies under these more challenging conditions and reporting full-cell performance metrics alongside half-cell data. Without such validation, the gap between laboratory breakthroughs and practical applications will remain substantial.

Despite these advances, a substantial gap remains before the practical implementation of SMAs. Looking ahead, we highlight the following key directions for future research:

(1) The dynamic evolution mechanisms and the precise regulation of the interfacial chemistry require further exploration. The current understanding of SEIs is largely based on ex situ or quasi-in situ characterizations, which fail to capture their real-time dynamic behavior under operating conditions. Future efforts should integrate in situ/operando spectroscopy, cryo-electron microscopy, and theoretical calculations to reveal the microscopic mechanisms of SEI nucleation, growth, cracking, and self-repair. This integration aims to achieve atomic-level control over their composition, structure, and thickness.
(2) The synergistic integration of multiple strategies and system-level optimization is essential. A single strategy often cannot simultaneously address all challenges faced by SMAs. The coordinated design of 3D current collectors, electrolyte additives, and artificial SEI layers can yield synergistic effects that surpass the sum of individual contributions. Moreover, the practical impact of each strategy should be systematically evaluated in full-cell configurations. This evaluation must consider engineering parameters, including the electrolyte-to-active-material ratio, the negative-to-positive capacity ratio, and the compatibility of the cathode.
(3) Breakthroughs in terms of high areal capacity and high-rate performance are crucial for practical applications. Most current studies are conducted under low areal capacities (<5 mAh cm^−2^) and low current densities (<5 mA cm^−2^), which are far from practical requirements (>5 mAh cm^−2^, >10 mA cm^−2^). Future research should focus on developing novel 3D hosts and composite anode architectures that exhibit high ionic conductivity, excellent mechanical properties, and the capability to accommodate substantial volume changes, while also verifying their reliability under harsh operating conditions.
(4) Advanced characterization and artificial intelligence-driven material discovery are essential for the progression of SMAs. By leveraging large-scale scientific facilities such as synchrotron radiation and neutron scattering, in conjunction with machine learning and high-throughput computation, the screening of novel electrolyte formulations, additive molecules, and SEI components can be significantly accelerated. This paradigm shift from trial-and-error methodologies to predictive design is expected to considerably shorten the research and development cycle for high-performance SMA materials.

In conclusion, while the commercialization of SMAs presents challenges, the prospects for developing stable, safe, and high-efficiency SMBs are promising, involving multidisciplinary integration and synergistic strategies. We believe that, as our understanding of the interfacial chemistry, deposition mechanisms, and failure origins deepens, SMAs will become indispensable in future large-scale energy storage systems.

## Figures and Tables

**Table 1:** Summary of representative strategies for stabilizing sodium metal anodes: key performance parameters, advantages, and limitations.

Strategy	Representative Example	CE	Work Condition of Symmetric Cells	Cycling Lifespan of Symmetric Cells	Major Advantages	Remaining Limitations	Ref.
Current collector engineering	Cu@Au	99.8% for 300 cycles at both 1.0 and 2.0 mA cm^−2^	-	-	Low nucleation overpotential; uniform dense deposition	Alloying/dealloying degrades sodiophilic layer over cycling	[46]
	Sn@C on Cu foil	~99.7% over 500 cycles at 2 mA cm^−2^ with 1 mAh cm^−2^	-	-	Carbon buffering layer mitigates volume change	Complex synthesis; limited areal capacity	[53]
	C@Sb on Cu foil	Average 99.7% for nearly 6000 h at 4 mAh cm^−2^	1 mA cm^−2^; 1 mAh cm^−2^	2400 h	Ultralong cycling; abundant nucleation sites	Sb core agglomeration over extended cycling	[54]
	3D porous Cu	99.4% after 400 cycles at 1 mA cm^−2^	1 mA cm^−2^; 1 mAh cm^−2^	1000 h	Large surface area; reduced local current density	Limited sodiophilicity; top-down deposition	[62]
	3D porous Al	99.8% over 1000 cycles at 1.0 mA cm^−2^ with 0.5 mAh cm^−2^	0.5 mA cm^−2^; 0.5 mAh cm^−2^	1000 h	Lightweight; cost-effective; uniform Na plating	Less explored compared to Cu-based current collectors	[64]
	D-ZnO NRAs/Al	Average CE of 99.95% at 1 mA cm^−2^ with 1 mAh cm^−2^ for 1500 cycles	1 mA cm^−2^; 1 mAh cm^−2^	2000 h	High sodiophilicity; regulates electric field	Scale-up of ZnO nanorod growth remains challenging	[73]
	Grad-CF (conductivity gradient)	99.85% for at least 150 cycles at 1 mA cm^−2^ with 5 mAh cm^−2^	2 mA cm^−2^; 4 mAh cm^−2^	320 h	Enables bottom-up deposition; suppresses dendrite penetration	Complex fabrication; gradient control precision	[89]
	Triple-gradient scaffold	Over 99.4% after 1200 cycles at 5 mAcm^−2^	5 mA cm^−2^; 5 mAh cm^−2^	1500 h	Integrates conductivity, sodiophilicity, and orientation gradients	Multistep preparation; high cost	[93]
Electrolyte engineering	1 M NaPF_6_ in G2	99.9% after 300 cycles at 0.5 mA cm^−2^	-	-	High CE; dendrite-free granular deposition	Ether solvents have limited oxidative stability	[103]
	1 M NaBF_4_ in TEGDME	99.9% over 1000 cycles at 0.5 mA cm^−2^ with 0.5 mAh cm^−2^	0.5 mA cm^−2^; 0.5 mAh cm^−2^	1000 h	Inorganic-rich uniform SEI	Lower ionic conductivity than carbonate electrolytes	[104]
	G2:MTHF co-solvent	99.71% over 300 cycles at 0.5 mA cm^−2^ with 1 mAh cm^−2^	0.5 mA cm^−2^; 1 mAh cm^−2^	Over 6000 h	CIP-dominated solvation; ultralong life	MTHF co-solvent increases cost and complexity	[113]
	KTFSI additive	Average CE of 99.5% at 0.5 mA cm^−2^ with 1 mAh cm^−2^ for 300 cycles	1 mA cm^−2^; 1 mAh cm^−2^	2700 h	Electrostatic shielding + SEI formation	Cation additive may complicate electrolyte formulation	[122]
Artificial SEI engineering	Na_3_P SEI	-	1 mA cm^−2^; 1 mAh cm^−2^	780 h	High ionic conductivity (0.12 mS cm^−1^); high Young’s modulus (8.6 GPa)	Thickness control difficult	[138]
	Na_2_Te SEI	-	1 mA cm^−2^; 1 mAh cm^−2^	700 h	Low Na^+^ migration barrier; high Young’s modulus (8.7 GPa)	Te precursor is relatively expensive	[139]
	Na_15_Sn_4_/Na_2_Se heterointerface	-	0.5 mA cm^−2^; 1 mAh cm^−2^	2400 h	Synergistic alloy + superionic conductor	Multistep fabrication; interface stability over long cycles	[141]
	Na_2_Se/V SEI	-	0.5 mA cm^−2^; 1 mAh cm^−2^	1790 h	Works in carbonate electrolytes; high sodiophilicity of V	Multistep fabrication	[142]
	IOHL (inorganic–organic)	-	1 mA cm^−2^; 1 mAh cm^−2^	770 h	High Young’s modulus (10.6 GPa); regulates Na^+^ distribution	Scalability of in situ reaction needs improvement	[152]
	PEO:SnF_2_ hybrid interface	-	0.5 mA cm^−2^; 0.25 mAh cm^−2^	760 h	Spontaneous conversion; dynamic self-reinforcing	Low current density; thickness control via drop-casting	[154]

## Data Availability

No new data were created or analyzed in this study. Data sharing is not applicable to this article.
